# (4-*tert*-Butyl­pyridine)­chlorido[hydro­tris­(3,5-dimethyl­pyrazol-1-yl)borato]nitro­sylmolybdenum(I) dichloro­methane monosolvate

**DOI:** 10.1107/S1600536810048233

**Published:** 2010-11-30

**Authors:** Mohammad B. Kassim, Jon A. McCleverty

**Affiliations:** a School of Chemistry, University of Bristol, Cantock Close, BS8 ITS Bristol, England

## Abstract

In the title compound, [Mo(C_15_H_22_BN_6_)Cl(NO)(C_9_H_13_N)]·CH_2_Cl_2_, the Mo^I^ atom adopts a distorted MoClN_5_ octa­hedral geometry with the hydro­tris­(3,5-dimethyl­pyrazol­yl)borate anion in an *N*,*N*′,*N*′′-tridentate tripodal (facial) coordination mode. A 4-*tert*-butyl­pyrine ligand, chloride anion and a nitrosyl cation complement the coodination of the Mo^I^ atom and an intra­molecular C—H⋯Cl hydrogen bond helps to stabilize the configuration of the complex mol­ecule. The packing is stabilized by an inter­molecular C—H⋯Cl hydrogen bond involving the complex mol­ecule and the CH_2_Cl_2_ solvent mol­ecule.

## Related literature

For bond lengths and angles, see: Kassim & McCleverty (2010 ▶). For related compounds, see: Kassim (2003 ▶); Kassim *et al.* (2002 ▶); Jones *et al.* (1997 ▶); Amoroso *et al.* (1994 ▶). For background to poly-(pyrazol­yl)borate ligands, see: Trofimenko (1993 ▶). For bond-length data, see: Allen *et al.* (1987 ▶). For the stability of the temperature controller used in the data collection, see: Cosier & Glazer (1986 ▶).
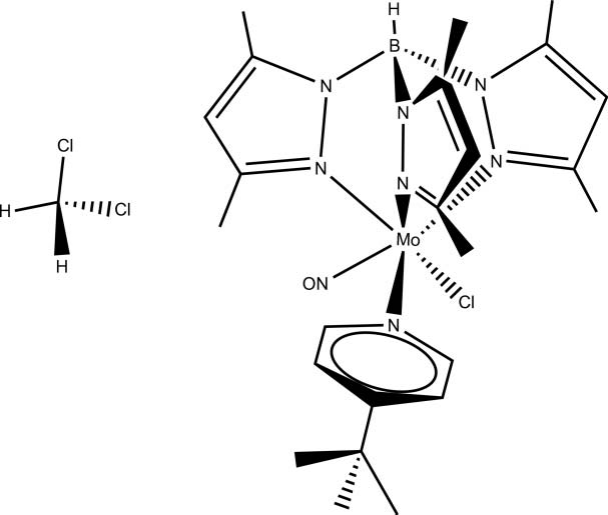


## Experimental

### 

#### Crystal data

[Mo(C_15_H_22_BN_6_)Cl(NO)(C_9_H_13_N)]·CH_2_Cl_2_
*M* *_r_* = 678.73
Monoclinic, 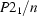

*a* = 13.4525 (18) Å
*b* = 16.345 (2) Å
*c* = 14.818 (2) Å
β = 109.376 (2)°
*V* = 3073.7 (7) Å^3^
*Z* = 4
Mo *K*α radiation
μ = 0.72 mm^−1^
*T* = 173 K
0.30 × 0.15 × 0.10 mm

#### Data collection

Bruker SMART APEX CCD diffractometer
Absorption correction: multi-scan (*SADABS*; Bruker, 2000 ▶) *T*_min_ = 0.878, *T*_max_ = 0.930
19465 measured reflections
7040 independent reflections
5113 reflections with *I* > 2σ(*I*)
*R*_int_ = 0.036

#### Refinement

*R*[*F* ^2^ > 2σ(*F* ^2^)] = 0.045
*wR*(*F* ^2^) = 0.119
*S* = 1.05
7040 reflections
365 parameters
H atoms treated by a mixture of independent and constrained refinement
Δρ_max_ = 1.54 e Å^−3^
Δρ_min_ = −1.50 e Å^−3^

### 

Data collection: *SMART* (Bruker, 2000 ▶); cell refinement: *SAINT* (Bruker, 2000 ▶); data reduction: *SAINT*; program(s) used to solve structure: *SHELXS97* (Sheldrick, 2008 ▶); program(s) used to refine structure: *SHELXL97* (Sheldrick, 2008 ▶); molecular graphics: *PLATON* (Spek, 2009 ▶) and *SHELXTL* (Sheldrick, 2008 ▶); software used to prepare material for publication: *PLATON*.

## Supplementary Material

Crystal structure: contains datablocks global, I. DOI: 10.1107/S1600536810048233/hb5750sup1.cif

Structure factors: contains datablocks I. DOI: 10.1107/S1600536810048233/hb5750Isup2.hkl

Additional supplementary materials:  crystallographic information; 3D view; checkCIF report

## Figures and Tables

**Table 1:** Selected bond lengths (Å)

Mo1—N1	1.999 (6)
Mo1—N21	2.164 (3)
Mo1—N11	2.184 (3)
Mo1—N41	2.207 (3)
Mo1—N31	2.248 (3)
Mo1—Cl1	2.4119 (14)

**Table 2:** Hydrogen-bond geometry (Å, °)

*D*—H⋯*A*	*D*—H	H⋯*A*	*D*⋯*A*	*D*—H⋯*A*
C36—H36*A*⋯Cl1	0.96	2.57	3.437 (5)	150
C51—H51*B*⋯Cl1^i^	0.97	2.48	3.412 (6)	161

Symmetry code: (i) 
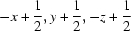
.

**Fig. 1.**
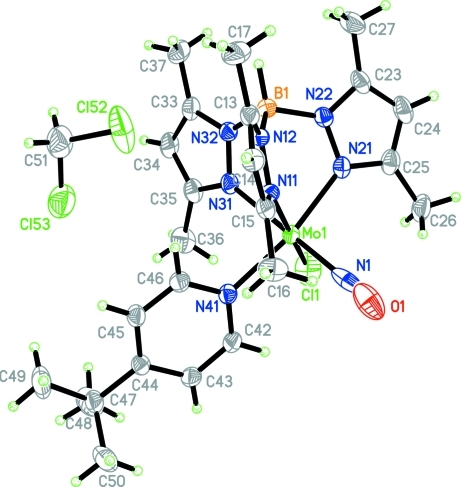
The title compound, (I), with displacement ellipsoids drawn at the 50% probability level. H atoms are shown as spheres of arbitary radius.

**Fig. 2.**
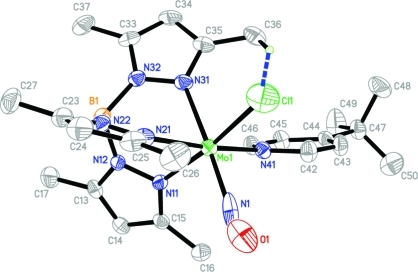
The complex molecule picturing the intramolecular H-bond shown as dotted line. The displacement ellipsoids are drawn at the 50% probability level and H atom is shown as spheres of arbitary radius.

**Fig. 3.**
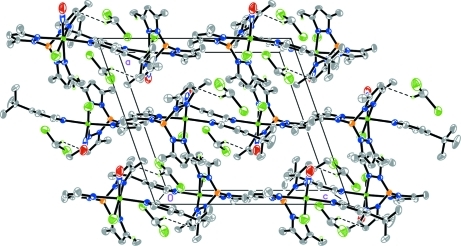
The packing diagram of the title compound, (I), view down the crystallographic b-axis showing the intermolecular H-bonds [symmetry code: -x + 1/2, y + 1/2,-z + 1/2]. The displacement ellipsoids are drawn at the 50% probability level and H atoms are shown as spheres of arbitary radius.

## supplementary crystallographic information

### Comment

Poly(pyrazolyl)borate ligands [Trofimenko (1993)] have attracted many researchers for the coordination chemistry of molybdenum complexes [Kassim *et al.* (2002), Jones *et al.* (1997) & Amoroso *et al.* (1994)]. In the title compound, (I), the hydrotris(3,5-dimethyl(pyrazolyl)borate ligand bonds to the central molybdenum atom in a tridentate manner through the N-atom at the 6-position of the pyrazolyl rings. One chloride anion; a 4-*tert*-butylpyridine and a nitrosyl cation, bond *via* the N-atom, establish the distorted octahedral coordination of the Mo(I) centre (Fig1). In addition, one molecule of CH_2_Cl_2_ solvent cystallized together with the complex molecule.

In the complex molecule moeities, [Mo1/Cl1/N11/N12/C13/C14/C15/C16/C17/B1 (A)], [Mo1/N21/N 22/C23/C24/C25/C26/C27/B1 (B)] and [Mo1/O1/N1/N31/N32/C33/C34/C35/C36/C37/B1 (C)] are essentially planar with maximum deviations from the mean plane are 0.040 (4)° for B1, 0.029 (5)° for C27 and 0.043 (1)° for B1 atoms, respectively. The dihedral angles between A/B, A/C and B/C planes are 62.18 (10)°, 56.96 (9)° and 60.87 (10)°, respectively. Whereas the dihedral angles between these moeities and the 4-*tert*-butylpyridine, [N41/C42/C43/C44/C45/C46/C47/C49 (D)] which is essentially planar with maximum deviation from the mean plane is 0.056 (4)° for C47 atom, are A/D 70.28 (13)°, B/D 18.14 (14)° and C/D 55.67 (13)°, respectively.

The crystal structure is stabilized by an intramolecular hydrogen bonds C(36)—H(36 A)···Cl(1) (Fig2). The crystal packing is stabilized by an intermolecular hydrogen bonds C—H···Cl (Fig3).

### Experimental

The title compound was synthesized from a reaction of Mo(NO)Tp*Cl_2_ (0.5 mmol) with 4- *tert*-butylpyridine (0.5 mmol) in dichloromethane in the presence of triethylammine at refluxing temperature under N_2_ atmosphere (Kassim 2003 & Kassim *et al.* 2002).  Green blocks of (I) were obtained from a slow evaporation of dichloromethane solution of the title compound at room temperature. Yeild 87%.

### Refinement

The H atoms attached to the B atom was located in a difference map, but those attached to carbon atoms were repositioned geometrically. The H atoms were initially refined with soft restraints on the bond lengths and angles to regularize their geometry (C—H in the range of 0.93–0.98, and O—H = 0.82 Å) and *U*_iso_(H) (in the range 1.2–1.5 times *U*_eq_ of the parent atom), after which the positions were refined with riding constraints.

### Crystal data

**Table:**

[Mo(C_15_H_22_BN_6_)Cl(NO)(C_9_H_13_N)]·CH_2_Cl_2_	*F*(000) = 1396
*M**_r_* = 678.73	*D*_x_ = 1.467 Mg m^−^^3^
Monoclinic, *P*2_1_/*n*	Mo *K*α radiation, λ = 0.71073 Å
Hall symbol: -P 2yn	Cell parameters from 7070 reflections
*a* = 13.4525 (18) Å	θ = 0.9–0.9°
*b* = 16.345 (2) Å	µ = 0.72 mm^−^^1^
*c* = 14.818 (2) Å	*T* = 173 K
β = 109.376 (2)°	Block, green
*V* = 3073.7 (7) Å^3^	0.30 × 0.15 × 0.10 mm
*Z* = 4	

### Data collection

**Table:**

Bruker SMART APEX CCD diffractometer	7040 independent reflections
Radiation source: fine-focus sealed tube	5113 reflections with *I* > 2σ(*I*)
graphite	*R*_int_ = 0.036
ω/2θ scans	θ_max_ = 27.5°, θ_min_ = 1.8°
Absorption correction: multi-scan (*SADABS*; Bruker, 2000)	*h* = −17→8
*T*_min_ = 0.878, *T*_max_ = 0.930	*k* = −20→21
19465 measured reflections	*l* = −17→19

### Refinement

**Table:**

Refinement on *F*^2^	Primary atom site location: structure-invariant direct methods
Least-squares matrix: full	Secondary atom site location: difference Fourier map
*R*[*F*^2^ > 2σ(*F*^2^)] = 0.045	Hydrogen site location: inferred from neighbouring sites
*wR*(*F*^2^) = 0.119	H atoms treated by a mixture of independent and constrained refinement
*S* = 1.05	*w* = 1/[σ^2^(*F*_o_^2^) + (0.0573*P*)^2^ + 2.0808*P*] where *P* = (*F*_o_^2^ + 2*F*_c_^2^)/3
7040 reflections	(Δ/σ)_max_ < 0.001
365 parameters	Δρ_max_ = 1.54 e Å^−^^3^
0 restraints	Δρ_min_ = −1.50 e Å^−^^3^

### Special details

**Table:**

Experimental. The crystal was placed in the cold stream of an Oxford Cryosystems open-flow nitrogen cryostat (Cosier & Glazer 1986) with a nominal stability of 0.1 K.
Geometry. All e.s.d.'s (except the e.s.d. in the dihedral angle between two l.s. planes) are estimated using the full covariance matrix. The cell e.s.d.'s are taken into account individually in the estimation of e.s.d.'s in distances, angles and torsion angles; correlations between e.s.d.'s in cell parameters are only used when they are defined by crystal symmetry. An approximate (isotropic) treatment of cell e.s.d.'s is used for estimating e.s.d.'s involving l.s. planes.
Refinement. Refinement of *F*^2^ against ALL reflections. The weighted *R*-factor *wR* and goodness of fit *S* are based on *F*^2^, conventional *R*-factors *R* are based on *F*, with *F* set to zero for negative *F*^2^. The threshold expression of *F*^2^ > σ(*F*^2^) is used only for calculating *R*-factors(gt) *etc*. and is not relevant to the choice of reflections for refinement. *R*-factors based on *F*^2^ are statistically about twice as large as those based on *F*, and *R*- factors based on ALL data will be even larger.

### Fractional atomic coordinates and isotropic or equivalent isotropic displacement parameters (Å^2^)

**Table:**

	*x*	*y*	*z*	*U*_iso_*/*U*_eq_	
Mo1	−0.01682 (2)	0.190933 (16)	0.201620 (19)	0.02194 (9)	
Cl1	0.09795 (11)	0.07454 (9)	0.25484 (10)	0.0659 (3)	
O1	−0.1941 (4)	0.0915 (3)	0.1352 (4)	0.0633 (14)	
N1	−0.1472 (5)	0.1227 (3)	0.1546 (4)	0.0506 (16)	
N11	−0.1070 (2)	0.30427 (15)	0.16343 (18)	0.0210 (5)	
N12	−0.0789 (2)	0.36951 (15)	0.22502 (18)	0.0209 (6)	
N21	−0.0271 (2)	0.21357 (17)	0.3421 (2)	0.0260 (6)	
N22	−0.0113 (2)	0.29123 (17)	0.37923 (19)	0.0246 (6)	
N31	0.1233 (2)	0.27485 (17)	0.25025 (19)	0.0257 (6)	
N32	0.1155 (2)	0.34300 (17)	0.30134 (19)	0.0244 (6)	
N41	0.0079 (2)	0.18807 (16)	0.06164 (19)	0.0238 (6)	
C13	−0.1469 (3)	0.43159 (19)	0.1912 (2)	0.0261 (7)	
C14	−0.2200 (3)	0.4062 (2)	0.1067 (2)	0.0275 (7)	
H14	−0.2764	0.4364	0.0676	0.033*	
C15	−0.1933 (3)	0.3268 (2)	0.0910 (2)	0.0239 (7)	
C16	−0.2479 (3)	0.2725 (2)	0.0085 (3)	0.0361 (9)	
H16A	−0.1985	0.2546	−0.0215	0.054*	
H16B	−0.3041	0.3021	−0.0369	0.054*	
H16C	−0.2761	0.2258	0.0310	0.054*	
C17	−0.1376 (3)	0.5122 (2)	0.2406 (3)	0.0406 (9)	
H17A	−0.1540	0.5057	0.2985	0.061*	
H17B	−0.1860	0.5504	0.1994	0.061*	
H17C	−0.0670	0.5324	0.2556	0.061*	
C23	−0.0187 (3)	0.2914 (2)	0.4679 (2)	0.0323 (8)	
C24	−0.0413 (3)	0.2125 (3)	0.4880 (3)	0.0389 (9)	
H24	−0.0513	0.1943	0.5438	0.047*	
C25	−0.0460 (3)	0.1657 (2)	0.4090 (3)	0.0334 (8)	
C26	−0.0657 (4)	0.0756 (2)	0.3935 (3)	0.0450 (10)	
H26A	−0.1189	0.0665	0.3325	0.068*	
H26B	−0.0892	0.0538	0.4432	0.068*	
H26C	−0.0017	0.0487	0.3950	0.068*	
C27	−0.0024 (4)	0.3667 (3)	0.5285 (3)	0.0457 (10)	
H27A	0.0710	0.3810	0.5506	0.069*	
H27B	−0.0250	0.3564	0.5825	0.069*	
H27C	−0.0427	0.4109	0.4914	0.069*	
C33	0.2073 (3)	0.3848 (2)	0.3266 (2)	0.0295 (8)	
C34	0.2748 (3)	0.3431 (2)	0.2915 (3)	0.0341 (8)	
H34	0.3435	0.3578	0.2979	0.041*	
C35	0.2212 (3)	0.2748 (2)	0.2446 (3)	0.0314 (8)	
C36	0.2608 (3)	0.2098 (3)	0.1939 (3)	0.0440 (10)	
H36A	0.2254	0.1592	0.1959	0.066*	
H36B	0.3352	0.2029	0.2247	0.066*	
H36C	0.2470	0.2257	0.1285	0.066*	
C37	0.2245 (3)	0.4632 (2)	0.3822 (3)	0.0411 (10)	
H37A	0.1683	0.5006	0.3518	0.062*	
H37B	0.2904	0.4869	0.3841	0.062*	
H37C	0.2257	0.4523	0.4462	0.062*	
C42	0.0214 (3)	0.1171 (2)	0.0216 (3)	0.0311 (8)	
H42	0.0079	0.0686	0.0483	0.037*	
C43	0.0542 (3)	0.1124 (2)	−0.0569 (3)	0.0301 (8)	
H43	0.0634	0.0615	−0.0811	0.036*	
C44	0.0738 (3)	0.1834 (2)	−0.1005 (2)	0.0253 (7)	
C45	0.0539 (3)	0.2565 (2)	−0.0615 (2)	0.0294 (8)	
H45	0.0619	0.3059	−0.0894	0.035*	
C46	0.0224 (3)	0.2567 (2)	0.0179 (2)	0.0284 (7)	
H46	0.0107	0.3067	0.0424	0.034*	
C47	0.1185 (3)	0.1786 (2)	−0.1822 (3)	0.0323 (8)	
C48	0.2272 (3)	0.1384 (3)	−0.1414 (3)	0.0549 (12)	
H48A	0.2618	0.1398	−0.1886	0.082*	
H48B	0.2192	0.0827	−0.1247	0.082*	
H48C	0.2688	0.1678	−0.0855	0.082*	
C49	0.1299 (5)	0.2623 (3)	−0.2217 (4)	0.0710 (17)	
H49A	0.1589	0.2565	−0.2725	0.106*	
H49B	0.1761	0.2954	−0.1717	0.106*	
H49C	0.0620	0.2880	−0.2459	0.106*	
C50	0.0491 (4)	0.1245 (3)	−0.2622 (3)	0.0522 (12)	
H50A	−0.0201	0.1481	−0.2873	0.078*	
H50B	0.0442	0.0709	−0.2375	0.078*	
H50C	0.0793	0.1206	−0.3124	0.078*	
C51	0.1685 (4)	0.5102 (3)	0.0856 (4)	0.0593 (13)	
H51A	0.1460	0.5490	0.0333	0.071*	
H51B	0.2240	0.5356	0.1375	0.071*	
Cl52	0.06149 (10)	0.48814 (11)	0.12480 (9)	0.0811 (5)	
Cl53	0.21867 (12)	0.42286 (8)	0.04752 (10)	0.0710 (4)	
B1	0.0133 (3)	0.3618 (2)	0.3211 (3)	0.0258 (8)	
H1	0.021 (2)	0.4210 (19)	0.360 (2)	0.016 (8)*	

### Atomic displacement parameters (Å^2^)

**Table:**

	*U*^11^	*U*^22^	*U*^33^	*U*^12^	*U*^13^	*U*^23^
Mo1	0.02209 (15)	0.02111 (14)	0.02357 (15)	−0.00110 (12)	0.00883 (11)	0.00138 (11)
Cl1	0.0607 (8)	0.0723 (8)	0.0686 (8)	−0.0042 (6)	0.0267 (7)	0.0112 (6)
O1	0.073 (3)	0.081 (4)	0.044 (3)	0.029 (2)	0.030 (2)	0.024 (2)
N1	0.106 (5)	0.029 (2)	0.029 (2)	0.036 (2)	0.039 (3)	0.0152 (18)
N11	0.0200 (13)	0.0219 (13)	0.0212 (13)	−0.0019 (11)	0.0071 (11)	−0.0018 (10)
N12	0.0203 (14)	0.0220 (13)	0.0224 (13)	−0.0034 (11)	0.0097 (11)	−0.0033 (10)
N21	0.0244 (15)	0.0284 (15)	0.0253 (14)	−0.0024 (11)	0.0087 (12)	0.0045 (11)
N22	0.0220 (15)	0.0313 (15)	0.0195 (13)	−0.0025 (11)	0.0055 (11)	−0.0001 (11)
N31	0.0199 (15)	0.0320 (15)	0.0261 (15)	−0.0033 (12)	0.0087 (12)	0.0007 (12)
N32	0.0233 (15)	0.0271 (14)	0.0226 (14)	−0.0034 (12)	0.0075 (12)	−0.0007 (11)
N41	0.0241 (14)	0.0217 (13)	0.0272 (14)	0.0014 (11)	0.0105 (12)	0.0019 (11)
C13	0.0289 (18)	0.0224 (16)	0.0329 (18)	0.0021 (14)	0.0181 (15)	0.0013 (13)
C14	0.0249 (18)	0.0273 (17)	0.0310 (18)	0.0056 (14)	0.0103 (15)	0.0040 (14)
C15	0.0208 (16)	0.0293 (18)	0.0238 (16)	0.0003 (13)	0.0105 (13)	0.0016 (12)
C16	0.028 (2)	0.042 (2)	0.0318 (19)	0.0042 (16)	0.0010 (16)	−0.0053 (16)
C17	0.041 (2)	0.0288 (19)	0.052 (2)	0.0030 (17)	0.0155 (19)	−0.0067 (17)
C23	0.0283 (19)	0.049 (2)	0.0201 (17)	−0.0032 (16)	0.0088 (15)	0.0009 (14)
C24	0.038 (2)	0.058 (3)	0.0223 (18)	−0.0022 (18)	0.0116 (16)	0.0103 (16)
C25	0.029 (2)	0.039 (2)	0.0291 (19)	−0.0049 (16)	0.0062 (15)	0.0095 (15)
C26	0.053 (3)	0.042 (2)	0.042 (2)	−0.014 (2)	0.018 (2)	0.0110 (18)
C27	0.052 (3)	0.059 (3)	0.028 (2)	−0.008 (2)	0.0166 (19)	−0.0121 (18)
C33	0.0238 (18)	0.038 (2)	0.0221 (17)	−0.0094 (15)	0.0012 (14)	0.0051 (14)
C34	0.0181 (18)	0.048 (2)	0.035 (2)	−0.0075 (16)	0.0071 (15)	0.0048 (16)
C35	0.0209 (18)	0.042 (2)	0.0311 (19)	0.0007 (15)	0.0085 (15)	0.0064 (15)
C36	0.025 (2)	0.057 (3)	0.054 (3)	0.0037 (18)	0.0183 (19)	−0.004 (2)
C37	0.039 (2)	0.043 (2)	0.038 (2)	−0.0185 (18)	0.0083 (18)	−0.0068 (17)
C42	0.039 (2)	0.0226 (18)	0.0367 (19)	−0.0040 (15)	0.0187 (17)	−0.0005 (14)
C43	0.035 (2)	0.0227 (17)	0.037 (2)	−0.0006 (15)	0.0183 (16)	−0.0036 (14)
C44	0.0223 (16)	0.0277 (17)	0.0278 (17)	0.0002 (14)	0.0108 (14)	−0.0001 (13)
C45	0.037 (2)	0.0235 (17)	0.0324 (19)	0.0017 (15)	0.0181 (16)	0.0053 (14)
C46	0.034 (2)	0.0216 (16)	0.0327 (18)	0.0025 (14)	0.0146 (16)	0.0006 (13)
C47	0.038 (2)	0.0307 (19)	0.037 (2)	−0.0018 (15)	0.0238 (17)	0.0000 (14)
C48	0.038 (3)	0.076 (3)	0.060 (3)	0.007 (2)	0.028 (2)	−0.006 (2)
C49	0.124 (5)	0.041 (3)	0.084 (4)	−0.003 (3)	0.083 (4)	0.007 (2)
C50	0.057 (3)	0.073 (3)	0.034 (2)	−0.012 (2)	0.025 (2)	−0.007 (2)
C51	0.046 (3)	0.076 (3)	0.056 (3)	−0.023 (2)	0.016 (2)	−0.001 (2)
Cl52	0.0496 (8)	0.1453 (14)	0.0461 (7)	−0.0186 (8)	0.0129 (6)	0.0341 (8)
Cl53	0.0898 (10)	0.0600 (8)	0.0600 (8)	−0.0163 (7)	0.0207 (7)	0.0117 (6)
B1	0.027 (2)	0.0265 (19)	0.0240 (19)	−0.0045 (15)	0.0088 (16)	−0.0033 (14)

### Geometric parameters (Å, °)

**Table:**

Mo1—N1	1.999 (6)	C27—H27A	0.9600
Mo1—N21	2.164 (3)	C27—H27B	0.9600
Mo1—N11	2.184 (3)	C27—H27C	0.9600
Mo1—N41	2.207 (3)	C33—C34	1.368 (5)
Mo1—N31	2.248 (3)	C33—C37	1.499 (5)
Mo1—Cl1	2.4119 (14)	C34—C35	1.385 (5)
O1—N1	0.787 (6)	C34—H34	0.9300
N11—C15	1.345 (4)	C35—C36	1.496 (5)
N11—N12	1.373 (3)	C36—H36A	0.9600
N12—C13	1.347 (4)	C36—H36B	0.9600
N12—B1	1.552 (5)	C36—H36C	0.9600
N21—C25	1.352 (4)	C37—H37A	0.9600
N21—N22	1.371 (4)	C37—H37B	0.9600
N22—C23	1.350 (4)	C37—H37C	0.9600
N22—B1	1.540 (5)	C42—C43	1.376 (5)
N31—C35	1.347 (4)	C42—H42	0.9300
N31—N32	1.371 (4)	C43—C44	1.395 (5)
N32—C33	1.351 (4)	C43—H43	0.9300
N32—B1	1.529 (5)	C44—C45	1.390 (5)
N41—C46	1.341 (4)	C44—C47	1.522 (5)
N41—C42	1.342 (4)	C45—C46	1.376 (5)
C13—C14	1.375 (5)	C45—H45	0.9300
C13—C17	1.492 (5)	C46—H46	0.9300
C14—C15	1.385 (5)	C47—C49	1.517 (5)
C14—H14	0.9300	C47—C50	1.525 (5)
C15—C16	1.493 (5)	C47—C48	1.532 (6)
C16—H16A	0.9600	C48—H48A	0.9600
C16—H16B	0.9600	C48—H48B	0.9600
C16—H16C	0.9600	C48—H48C	0.9600
C17—H17A	0.9600	C49—H49A	0.9600
C17—H17B	0.9600	C49—H49B	0.9600
C17—H17C	0.9600	C49—H49C	0.9600
C23—C24	1.380 (5)	C50—H50A	0.9600
C23—C27	1.496 (5)	C50—H50B	0.9600
C24—C25	1.382 (5)	C50—H50C	0.9600
C24—H24	0.9300	C51—Cl53	1.751 (5)
C25—C26	1.501 (5)	C51—Cl52	1.758 (5)
C26—H26A	0.9600	C51—H51A	0.9700
C26—H26B	0.9600	C51—H51B	0.9700
C26—H26C	0.9600	B1—H1	1.12 (3)
			
N1—Mo1—N21	95.86 (16)	C23—C27—H27C	109.5
N1—Mo1—N11	92.03 (16)	H27A—C27—H27C	109.5
N21—Mo1—N11	84.08 (10)	H27B—C27—H27C	109.5
N1—Mo1—N41	92.79 (16)	N32—C33—C34	107.7 (3)
N21—Mo1—N41	170.21 (10)	N32—C33—C37	123.0 (3)
N11—Mo1—N41	91.05 (9)	C34—C33—C37	129.4 (3)
N1—Mo1—N31	176.27 (16)	C33—C34—C35	107.0 (3)
N21—Mo1—N31	84.37 (10)	C33—C34—H34	126.5
N11—Mo1—N31	84.28 (10)	C35—C34—H34	126.5
N41—Mo1—N31	86.70 (10)	N31—C35—C34	108.9 (3)
N1—Mo1—Cl1	93.79 (15)	N31—C35—C36	123.4 (3)
N21—Mo1—Cl1	93.37 (8)	C34—C35—C36	127.7 (3)
N11—Mo1—Cl1	173.86 (8)	C35—C36—H36A	109.5
N41—Mo1—Cl1	90.63 (8)	C35—C36—H36B	109.5
N31—Mo1—Cl1	89.92 (8)	H36A—C36—H36B	109.5
O1—N1—Mo1	173.1 (9)	C35—C36—H36C	109.5
C15—N11—N12	107.0 (2)	H36A—C36—H36C	109.5
C15—N11—Mo1	134.3 (2)	H36B—C36—H36C	109.5
N12—N11—Mo1	118.68 (19)	C33—C37—H37A	109.5
C13—N12—N11	109.5 (3)	C33—C37—H37B	109.5
C13—N12—B1	129.8 (3)	H37A—C37—H37B	109.5
N11—N12—B1	120.5 (3)	C33—C37—H37C	109.5
C25—N21—N22	106.5 (3)	H37A—C37—H37C	109.5
C25—N21—Mo1	134.0 (3)	H37B—C37—H37C	109.5
N22—N21—Mo1	119.55 (19)	N41—C42—C43	123.4 (3)
C23—N22—N21	109.9 (3)	N41—C42—H42	118.3
C23—N22—B1	129.9 (3)	C43—C42—H42	118.3
N21—N22—B1	120.2 (3)	C42—C43—C44	120.5 (3)
C35—N31—N32	106.9 (3)	C42—C43—H43	119.8
C35—N31—Mo1	135.5 (2)	C44—C43—H43	119.8
N32—N31—Mo1	117.6 (2)	C45—C44—C43	115.5 (3)
C33—N32—N31	109.4 (3)	C45—C44—C47	123.9 (3)
C33—N32—B1	130.0 (3)	C43—C44—C47	120.6 (3)
N31—N32—B1	120.5 (3)	C46—C45—C44	120.9 (3)
C46—N41—C42	116.5 (3)	C46—C45—H45	119.5
C46—N41—Mo1	121.9 (2)	C44—C45—H45	119.5
C42—N41—Mo1	121.2 (2)	N41—C46—C45	123.1 (3)
N12—C13—C14	107.7 (3)	N41—C46—H46	118.4
N12—C13—C17	123.1 (3)	C45—C46—H46	118.4
C14—C13—C17	129.2 (3)	C49—C47—C44	112.1 (3)
C13—C14—C15	106.8 (3)	C49—C47—C50	109.7 (4)
C13—C14—H14	126.6	C44—C47—C50	110.3 (3)
C15—C14—H14	126.6	C49—C47—C48	109.4 (4)
N11—C15—C14	109.0 (3)	C44—C47—C48	106.7 (3)
N11—C15—C16	123.4 (3)	C50—C47—C48	108.5 (3)
C14—C15—C16	127.6 (3)	C47—C48—H48A	109.5
C15—C16—H16A	109.5	C47—C48—H48B	109.5
C15—C16—H16B	109.5	H48A—C48—H48B	109.5
H16A—C16—H16B	109.5	C47—C48—H48C	109.5
C15—C16—H16C	109.5	H48A—C48—H48C	109.5
H16A—C16—H16C	109.5	H48B—C48—H48C	109.5
H16B—C16—H16C	109.5	C47—C49—H49A	109.5
C13—C17—H17A	109.5	C47—C49—H49B	109.5
C13—C17—H17B	109.5	H49A—C49—H49B	109.5
H17A—C17—H17B	109.5	C47—C49—H49C	109.5
C13—C17—H17C	109.5	H49A—C49—H49C	109.5
H17A—C17—H17C	109.5	H49B—C49—H49C	109.5
H17B—C17—H17C	109.5	C47—C50—H50A	109.5
N22—C23—C24	107.5 (3)	C47—C50—H50B	109.5
N22—C23—C27	122.9 (3)	H50A—C50—H50B	109.5
C24—C23—C27	129.7 (3)	C47—C50—H50C	109.5
C23—C24—C25	106.6 (3)	H50A—C50—H50C	109.5
C23—C24—H24	126.7	H50B—C50—H50C	109.5
C25—C24—H24	126.7	Cl53—C51—Cl52	112.7 (3)
N21—C25—C24	109.5 (3)	Cl53—C51—H51A	109.1
N21—C25—C26	121.5 (3)	Cl52—C51—H51A	109.1
C24—C25—C26	129.0 (3)	Cl53—C51—H51B	109.1
C25—C26—H26A	109.5	Cl52—C51—H51B	109.1
C25—C26—H26B	109.5	H51A—C51—H51B	107.8
H26A—C26—H26B	109.5	N32—B1—N22	109.3 (3)
C25—C26—H26C	109.5	N32—B1—N12	109.7 (3)
H26A—C26—H26C	109.5	N22—B1—N12	108.6 (3)
H26B—C26—H26C	109.5	N32—B1—H1	109.8 (16)
C23—C27—H27A	109.5	N22—B1—H1	110.9 (16)
C23—C27—H27B	109.5	N12—B1—H1	108.5 (16)
H27A—C27—H27B	109.5		

### Hydrogen-bond geometry (Å, °)

**Table:**

*D*—H···*A*	*D*—H	H···*A*	*D*···*A*	*D*—H···*A*
C36—H36A···Cl1	0.96	2.57	3.437 (5)	150
C51—H51B···Cl1^i^	0.97	2.48	3.412 (6)	161

Symmetry codes:  (i) −*x*+1/2, *y*+1/2, −*z*+1/2.
